# **If Left Unchecked:** Lessons Learned from Unfettered U.S. Government Support of the NIH-Moderna Vaccine

**DOI:** 10.1017/jme.2023.155

**Published:** 2023

**Authors:** Reshma Ramachandran

**Keywords:** COVID-19 Vaccines, Government Financing, Public Investment, Pharmaceutical Prices, Vaccines

## Abstract

The NIH-Moderna mRNA COVID-19 vaccine’s steep price increase raises concerns that this will be the new anchor for continued price hikes and underscores the need for upstream government intervention to enable greater accountability and stewardship of public biomedical research investment.

On January 10, 2023, in response to “widespread anger” over Moderna’s announcement that they planned to raise the price of its marketed mRNA COVID-19 vaccine by nearly 400%, the company’s CEO Stéphane Bancel was called to testify before the Senate Health, Education, Labor, and Pensions (HELP) Committee.^1^ In his remarks and responses to questions from Committee members, Bancel put forward an elaborate defense of Moderna’s plans.^2^ Immediately after, the HELP Committee convened a second panel of expert witnesses, including Dr. Ameet Sarpatwari, to ask the critical question of why Moderna would take such action despite major contributions from National Institutes of Health (NIH) scientists and billions in American taxpayer funding directed toward the development, and production of the vaccine.

In his testimony and article in this issue, “Public Returns on Public Investment: Moderna’s Violation of the Social Contract,” Sarpatwari systematically rebuts Moderna’s justification for the planned price increase of the NIH-Moderna vaccine and outlines in extensive detail how Moderna, a once fledgling company without a product previously authorized or approved by the U.S. Food and Drug Administration (FDA), benefitted from the federal government’s extraordinary investment and resources. As Sarpatwari illustrates, despite having received this critical public support, Moderna has effectively turned its back on American taxpayers in proceeding with its action.

This Commentary will elaborate on Sarpatwari’s refutation of Moderna’s arguments and recommended actions for the federal government to consider toward ensuring greater accountability and stewardship of public investment.

## Dismantling Moderna’s Rationale for the Planned Price Increase

Sarpatwari describes Moderna’s justification for the price increase of NIH-Moderna vaccine as threefold; namely that 1) such a price increase would be commensurate with the value of the vaccine; 2) the higher price would enable further investment into Moderna’s ongoing research and development; and 3) the price increase would be mitigated by Moderna’s planned launch of a patient assistance program and the lack of out-of-pocket payments for patients with insurance coverage. However, as detailed in Sarpatwari’s article, this rationale put forward by Moderna to defend their hefty price increase is disingenuous and flawed.

First, his careful documentation of the extraordinary public investment made by the federal government that enabled the successful development of the NIH-Moderna mRNA vaccine undermines the argument that Moderna should be allowed to extract the value of the vaccine through higher prices.^3^ Not only did Moderna receive significant direct public investment to accelerate the development of mRNA vaccines before and during the pandemic, but it also received additional resources in the form of technical expertise from close collaboration with government scientists and access to NIH clinical trial networks to successfully recruit trial participants representative of the US population.^4^ Moreover, the federal government all but ensured the success of the vaccine, even ahead of U.S. Food and Drug Administration (FDA) authorization through advanced purchasing agreements, securing hundreds of millions of doses at a price well above the cost of production.^5^ Thus, as Sarpatwari argues, the federal government bore both the cost of this development and the risk, mitigating the need for Moderna to raise, much less quadruple vaccine prices.

We found that between 2000 and 2021, average prices for the influenza vaccine rose by 149% for the public sector and 163% for the private sector, seemingly unaffected by the number of manufacturers or products on the market. Thus, it is likely that the current public and private sector procurement prices negotiated by Moderna could serve as starting point for continually rising prices should the vaccine continue to be recommended annually.

Besides these financial and other resources detailed by Sarpatwari that considerably “de-risked” the development of the NIH-Moderna vaccine, Moderna also received additional regulatory incentives with full FDA approval, well after being granted initial emergency use authorization and multiple advance market commitments. One such regulatory incentive was the medical countermeasure priority review voucher, awarded by the FDA to manufacturers of medical products that treat or prevent harm from material threats such as COVID-19.^6^ Redemption of these vouchers enables products that would have otherwise been ineligible to receive “priority review” shortening regulatory review ahead of market entry from a standard 10 months to 6 months.^7^ Unnecessary given the significant public investment and resources in addition to guaranteed revenues through several public procurement agreements,^8^ this estimated $100 million innovation incentive could either be sold by Moderna to another manufacturer or redeemed by Moderna itself for another product in its portfolio to hasten market entry.^9^ Thus, the public incentives received by Moderna go well beyond the NIH-Moderna vaccine alone, potentially impacting their larger product portfolio.

Sarpatwari also effectively counters the claim that such a price increase is necessary as Moderna will require further funds to support ongoing research and development. Since the initial FDA emergency use authorization in December 2020, Moderna has collected over $20 billion in profit leading to rising share prices and has catapulted Mr. Bancel into the billionaire class.^10^ Sarpatwari highlights how despite these large returns, Moderna did not prioritize investment into research and development, instead spending these funds largely on share buybacks meant to boost their share price and increase shareholder payouts.^11^ While Moderna focused its profit reinvestment strategy toward maximizing shareholder value, the company continued to collaborate closely with the NIH in developing variant-specific COVID-19 mRNA vaccines, again utilizing their technical expertise and established clinical trial sites.^12^ US government collaboration and investment have persisted in playing a significant role to de-risk further COVID-19 vaccine research and enable continued innovation for Moderna, further diminishing the need for raising prices.

## Public Health Ramifications and Opportunity Costs of Moderna’s Price Increase

Unfortunately, the public health ramifications of Moderna’s announcement to raise the price of the NIH-Moderna COVID-19 vaccine as described by Sarpatwari in his article are no longer theoretical. Shortly after the Centers for Disease Control and Prevention issued its recommendation that everyone 6 months and older get an updated COVID-19 vaccine this fall and winter,^13^ public and private sector costs for recommended COVID-19 mRNA vaccines were posted on their website. While private sector costs of $128 per dose for the COVID-19 vaccine for those age 12 years and older reflect Moderna’s earlier announcements, the public sector price is lower at $81.61 per dose.^14^ Still, this is approximately a 214% increase from the $26 public procurement price for the prior bivalent formulation of the vaccine and a 410% increase from $16 initial monovalent version.

Our team’s prior analysis public and private sector pricing trends of the influenza vaccine also foreshadows a sobering future for COVID-19 mRNA vaccine prices.^15^ Similar to the NIH-Moderna vaccine, influenza vaccines were discovered, developed, and manufactured with support from the public. As these vaccines are updated and administered annually, the federal government has played a continued collaborative role both in terms of clinical testing and manufacturing of updated vaccines as well as through public procurement of doses. We found that between 2000 and 2021, average prices for the influenza vaccine rose by 149% for the public sector and 163% for the private sector, seemingly unaffected by the number of manufacturers or products on the market. Thus, it is likely that the current public and private sector procurement prices negotiated by Moderna could serve as starting point for continually rising prices should the vaccine continue to be recommended annually.

As Sarpatwari notes, Moderna’s placations that Americans with insurance coverage will not face out-of-pocket payments for the vaccine fail to alleviate the public health ramifications of health plans having to shoulder these increased costs. Current and future higher prices for these vaccines may likely crowd out other critical expenditures to bolster core infrastructure from public health program budgets. These higher costs may also be transferred to insurance beneficiaries, regardless of whether they received the vaccine, in the form of higher coverage premiums. Sarpatwari also reveals the shortcomings of Moderna’s proposed patient assistance program to provide vaccines to those who are under- or uninsured, noting the difficulty in enrolling in such programs,^16^ which likely will lead to significant attrition in vaccine administration if it is the only program available for these populations.

Moderna announced this patient assistance program earlier this year and it was of significant interest to legislators during the March 2023 Senate HELP hearing.^16^ However, since this initial announcement, further details of the program including when it would be launched are not yet available. This may be due to the recent establishment of the CDC Bridge Access Program, which will provide no-cost COVID-19 vaccines to under- and uninsured adults until December 31, 2024.^17^ Presumably, CDC would have procured doses for this program from Moderna at the negotiated public sector cost of $81.61, allowing Moderna to avoid timely establishment of the patient assistance program through which doses would be provided, free of charge. With the establishment of this public procurement program from which Moderna can capture yet another sizeable federal subsidy, what incentive does the company have to directly provide free vaccines to uninsured patients?

## Policy Interventions to Ensure Accountability and Stewardship of Taxpayer Investment

Sarpatwari outlines several necessary policy interventions to prevent Moderna from price gouging the federal government and American for the publicly funded and developed vaccine. He recommends that Congress continue to place further pressure on the company to backtrack on its proposed price increase as such public scrutiny has been shown to be effective. On July 13, 2023, Department of Health and Human Services (HHS) Secretary Xavier Becerra publicly reminded COVID-19 vaccine manufacturers including Moderna that the “U.S. government has invested billions of dollars in research, development, and procurement for COVID-19 vaccines” and “federally-sponsored research has provided crucial insights that laid the groundwork for the development of COVID-19 vaccines over several decades”, urging them to not engage in price gouging behavior and set their prices at a “reasonable rate.”^18^ It is unclear whether this may have had an impact on the public sector price ultimately negotiated, a 34-59% decrease from Moderna’s proposed increase price.

Nevertheless, the Biden administration could do more to heighten public awareness of Moderna’s violation of the social contract. On February 7, 2023, President Biden in his State of the Union remarks called for capping the cost of insulin for all Americans building on provisions passed as part of the Inflation Reduction Act, which cap the cost of insulin for Medicare beneficiaries.^19^ Shortly after, insulin manufacturers announced significant price cuts to some of their insulin products.^20^ Although increased public pressure may move Moderna to lower their prices, the Biden administration could also further exercise their leverage when financing further development and procurement by requiring reasonable pricing as part of their contracts with drug manufacturers. Recent announcements of the administration successfully negotiating such provisions as part of their procurement contracts with COVID-19 drug and vaccine manufacturers have demonstrated their capability in doing so with little concern from both parties that such actions would have an undue impact on innovation. In late 2021, the US government procured 10 million doses of Paxlovid from Pfizer under the condition that the price per dose is either matches or is less than the lowest price negotiated among six other wealthy countries.^21^ In September, HHS announced similar such provisions in a more upstream investment agreement with Regeneron for development of a COVID-19 monoclonal antibody that would tether the price of the treatment to that of other selected high-income countries.^22^ Just recently, HHS announced that this “fair pricing” provision is now standard in all medical product development and procurement contracts. ^23^

The federal government will continue to invest in the development of new vaccines and other health technologies necessary to prepare for and respond to future public health threats through existing and new initiatives such as Project NextGen or the Advanced Research Projects Agency for Health (ARPA-H). Moving forward, the Biden administration could instead consider creating a public option for biomedical research and development instead of playing a collaborative role with the private sector, leaving control of supply, price, and scientific knowledge in the hands of private actors whose profit motivations will likely continue to remain misaligned with public health interests. As Sarpatwari illustrates, the public health ramifications of Moderna’s violation of their social contract to American taxpayers are immense. However, the lessons learned from this extraordinary public achievement can also offer a better path forward toward enabling greater accountability and stewardship of public funding for biomedical research.

## Note

Dr. Ramachandran is the Board President of Universities Allied for Essential Medicines North America and the Chair of the Doctors for America FDA Task Force. She has received a grant from the Swedish International Development and Cooperation Agency for participation in the ReAct-Action on Antibiotic Resistant Strategic Policy Program at Johns Hopkins Bloomberg School of Public Health.
